# Autoimmune Polyglandular Syndrome Type 2 With Hurthle Cell Adenoma: A Rare Association

**DOI:** 10.7759/cureus.92931

**Published:** 2025-09-22

**Authors:** Shalini S Pandya, Kush Shah, Hitesh Chavda, Kanan Shah

**Affiliations:** 1 Department of Internal Medicine, Sterling Hospitals, Ahmedabad, IND; 2 Department of Surgical Oncology, Kusum Dhirajlal (KD) Hospital, Ahmedabad, IND; 3 Department of Surgical Gastroenterology, Sterling Hospitals, Ahmedabad, IND; 4 Department of Dermatology, Sterling Hospitals, Ahmedabad, IND

**Keywords:** autoimmune polyglandular syndrome type 2 (aps 2), autoimmune thyroiditis, endocrine neoplasms, hashimoto’s thyroiditis, hurthle cell adenomas, primary adrenal insufficiency, schmidt syndrome, thyroid nodule

## Abstract

Autoimmune polyglandular syndrome type 2 (APS 2) is a rare endocrinopathy characterized by primary adrenal insufficiency associated with autoimmune thyroiditis or type 1 diabetes. The diagnosis is usually delayed, and it has high mortality if undetected. Hashimoto’s thyroiditis is the commonest thyroid disease associated with APS 2, while structural thyroid abnormalities or neoplasms are rarely reported. Hurthle cell adenoma (HCA) is a rare benign oncocytic tumor and may arise from long-standing chronic autoimmune thyroiditis. To date, there is no documented case of APS 2 with Hurthle cell adenoma, making this case a rarity.

We present a case of a 44-year-old man with gastrointestinal symptoms, fatigue, hyperpigmentation, and weight loss for six months. The critical findings were generalized hyperpigmentation with orthostatic hypotension and electrolyte imbalance, especially hyponatremia and hyperkalemia, with low cortisol and elevated adrenocorticotropic hormone levels. These, along with bilateral adrenal atrophy on imaging and high anti-thyroid peroxidase antibodies, established a diagnosis of primary adrenal insufficiency and autoimmune thyroiditis, findings consistent with APS 2. HCA was detected on fine needle biopsy subsequently, which is a rare association in APS 2. The patient responded well to adrenal and thyroxine replacement therapy. The patient’s tumor, although small and asymptomatic, was excised via hemithyroidectomy as per the American Thyroid Association guidelines. Histopathology confirmed HCA with no capsular invasion. Postoperative recovery was uneventful, and on follow-up, the patient showed significant improvement.

There are two major learning points from this case. Firstly, it emphasizes the need for high clinical suspicion in diagnosing APS 2 in patients with nonspecific constitutional symptoms. Secondly, it highlights the rare but clinically significant co-occurrence of HCA with APS 2. HCA, while benign, can mimic malignancy on cytology and requires histopathological confirmation. In view of the possibility of progression of HCA to Hurthle cell carcinoma, surgical excision is the standard of care.

## Introduction

Autoimmune polyglandular syndrome type 2 (APS 2) is a rare, potentially life-threatening syndrome if untreated and is defined by the presence of primary adrenal insufficiency in combination with autoimmune thyroid disease and/or type 1 diabetes mellitus [1,2]. It was first described in 1926 by Martin Benno Schmidt and so is also known as Schmidt syndrome. It is an immune-mediated destruction that affects two or more endocrine glands and causes multiple gland insufficiencies. It can be associated with vitiligo, pernicious anemia, chronic autoimmune hepatitis, alopecia, myasthenia gravis, rheumatoid arthritis, Sjogren’s syndrome, hypergonadotropic hypogonadism, and thrombocytopenic purpura [1,2]. The diagnosis is often delayed or missed because of its rarity, nonspecific symptoms, and delayed development of individual components of the syndrome. Patients often present with one endocrine disease, such as autoimmune thyroiditis or primary adrenal insufficiency, initially and may not develop the full picture of APS 2 for decades. Therefore, lifelong surveillance for associated diseases is recommended in these patients.

APS 2 is common in women (3:1) between the ages of 20 and 40 years and is strongly associated with the class II human leukocyte antigen (HLA) haplotypes DR3 and DR4. The prevalence of APS 2 is 1.4-2 per 100,000 population [1-3].

The most common thyroid pathology in APS 2 is Hashimoto’s thyroiditis, which often has elevated thyroid autoantibodies [1-3]. Structural thyroid abnormalities such as nodules or neoplasms are not routinely encountered in APS 2, and their occurrence warrants further investigation. Hurthle cell adenoma (HCA) is a rare benign thyroid adenoma comprising more than 75% oncocytic cells. It accounts for almost 5% of all thyroid tumors. The oncocytic cells are large polygonal cells with eosinophilic, granular cytoplasm due to abundant mitochondria and large nuclei with prominent nucleoli. It is essential to identify and excise them, as 30% of these adenomas are known to progress to aggressive Hurthle cell carcinoma [4].

Here, we report a unique case of APS 2 presenting with primary adrenal insufficiency and autoimmune thyroiditis, which, on further evaluation, revealed an HCA, ultimately managed with hemithyroidectomy. To our knowledge, this is the first documented case of APS 2 with HCA.

## Case presentation

A 44-year-old gentleman presented to the gastrosurgeon with increased frequency of semisolid stools for six months. There was no fever, bleeding per rectum, or abdominal pain. He also complained of anorexia, progressive hyperpigmentation of the skin, and generalized weakness, and had lost approximately 10 kg of weight in the last six months. Four days before presentation, he had 3-4 vomitings daily and was unable to eat. There was nothing significant in the past history. As his ultrasonography of the abdomen had been normal, he was referred to the department of internal medicine for further management.

**Figure 1:**
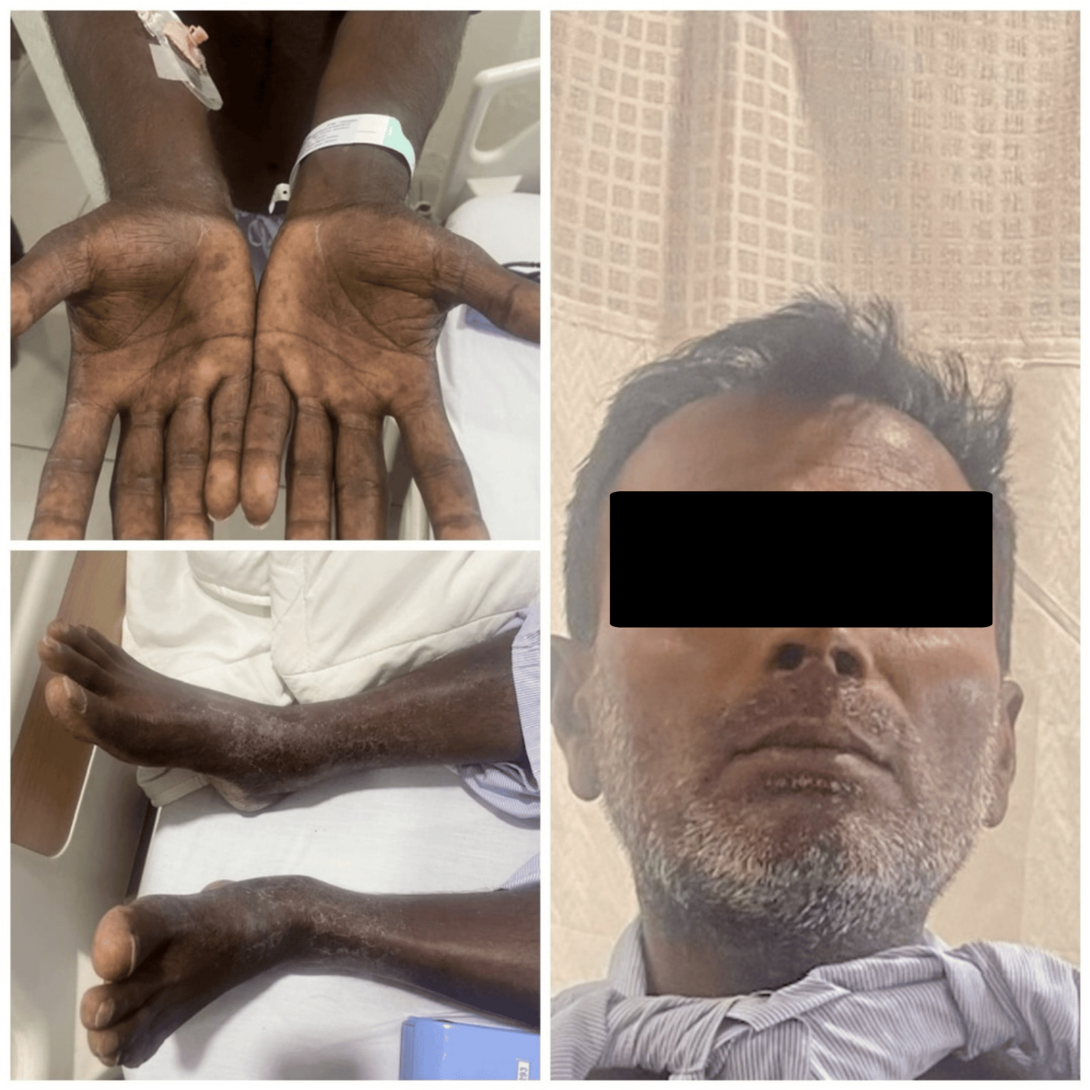
Patient profile on admission The patient had generalized hyperpigmentation, darkening of creases on the palms of the hand, and a cachectic look.

On admission, he had borderline low blood pressure with orthostatic hypotension and tachycardia, and he appeared dehydrated. On general examination, he had hyperpigmented, dry skin, a firm, mobile 2x1.5 cm nodule in the lower part of the right lobe of the thyroid gland with no lymphadenopathy (Figure 1). He had no signs suggestive of either hyper- or hypothyroidism. The rest of the systemic examination had been unremarkable.

He was started on intravenous fluids. The routine investigations were normal, except for borderline low blood sugars, hyponatremia, mild hyperkalemia, and borderline high thyroid-stimulating hormone (Table 1). On further investigation, he was found to have low cortisol, high adrenocorticotropic hormone, low aldosterone with normal plasma renin levels, and high anti-thyroid peroxidase antibodies (Table 1).

**Table 1:** Relevant investigations ACTH: adrenocorticotropic hormone (pg/mL), TSH: thyroid-stimulating hormone (IU/mL), total T3: total triiodothyronine (ng/mL), total T4: total thyroxine (mcg/dL), anti-TPO antibodies: anti-thyroid peroxidase antibodies (IU/mL)

Investigation	Values	Normal range
Serum creatinine	0.6 mg/dL	0.5-1.2 mg/dL
Serum sodium	127 mEq/L	135-145 mEq/L
Serum potassium	5.3 mEq/L	3.6-5.2 mEq/L
Serum cortisol	0.8 mcg/dL	3.7-19.4 mcg/dL
ACTH	>1,250 pg/mL	9-46 pg/mL
Serum aldosterone	<0.97 ng/dL	1.76-23.2 ng/dL
Plasma renin activity	1.98 ng/mL/hour	0.15-2.33 ng/mL/hour
TSH	9.6 IU/mL	0.5-4.5 IU/mL
Total T3	1 ng/mL	0.5-1.59 ng/mL
Total T4	9.63 mcg/dL	4.8-11.7 mcg/dL
Anti-TPO antibodies	379.45 IU/mL	<5.61 IU/mL
Tissue transglutaminase antibody	<0.2 U/mL	<8 U/mL

**Figure 2:**
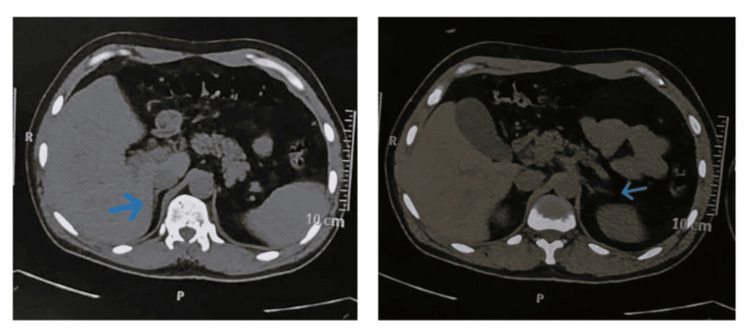
Computed tomography of the abdomen The computed tomography of the abdomen shows reduced volume of bilateral adrenal glands (arrows), suggestive of bilateral adrenal atrophy.

The ultrasonography of the thyroid gland revealed early changes of thyroiditis and a focal isoechoic capsulated lesion in the right lower lobe of the thyroid gland with insignificant reactive lymph nodes, suggestive of thyroiditis with parathyroid adenoma. The computed tomography of the abdomen showed bilateral adrenal atrophy (Figure 2).

**Figure 3:**
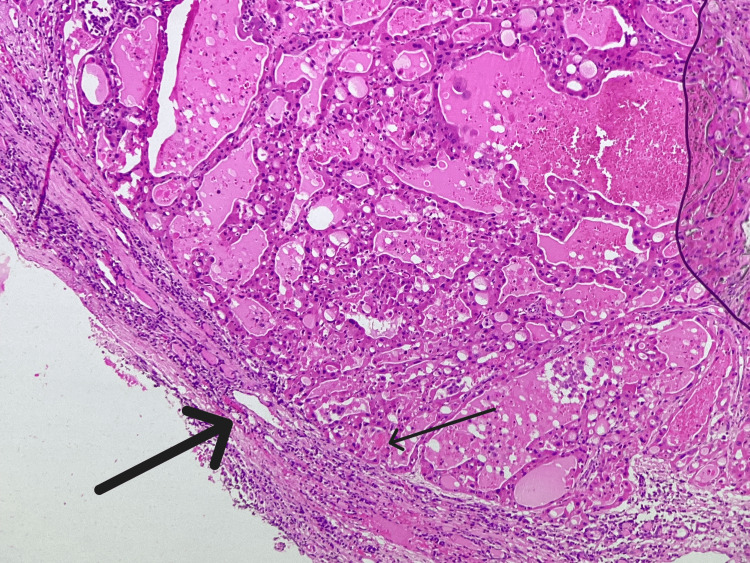
Histopathological section of the thyroid nodule (hematoxylin and eosin stain, 10× magnification) A well-defined capsule is seen (thick arrow), suggestive of adenoma along with Hurthle cells (thin arrow), characterized by a large polygonal shape, abundant granular eosinophilic cytoplasm, centrally placed nuclei, prominent nucleoli, and loss of cell polarity.

As calcium, phosphorus, and parathyroid hormone were in normal range, a diagnosis of primary adrenal insufficiency with autoimmune thyroiditis was made. He was initially given a hydrocortisone loading dose of 100 mg intravenously, followed by 50 mg every six hours for the first 48 hours, and then thyroxine replacement was started. He was symptomatically better in 48 hours, and orthostatic hypotension resolved. A fine needle aspiration biopsy showed HCA. The patient underwent hemithyroidectomy, and the histopathology report confirmed HCA (Figure 3).

**Figure 4:**
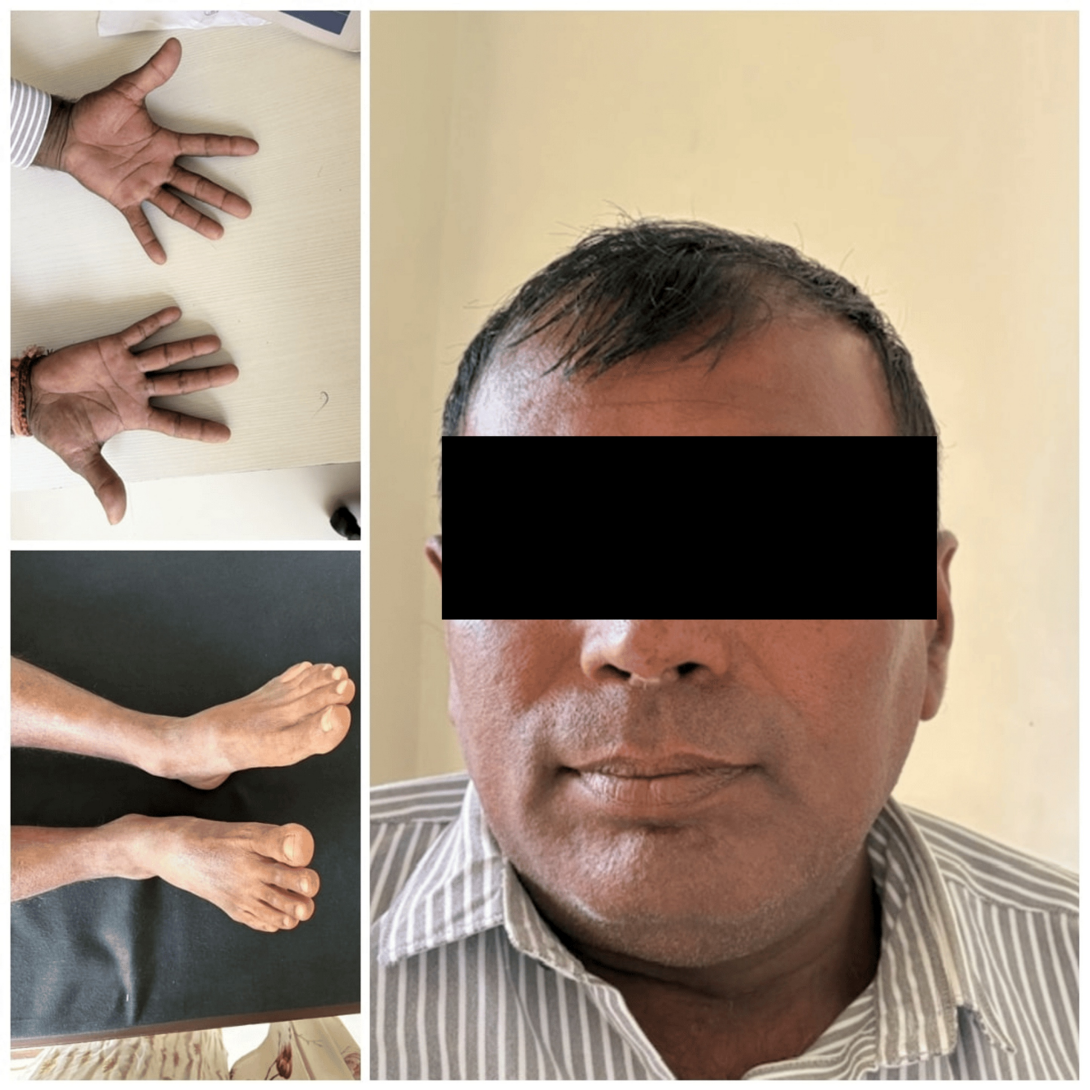
Patient at three-month follow-up The patient has gained weight, and the hyperpigmentation of the skin has reduced.

Further hospital course had been uneventful, and he was discharged on prednisolone, fludrocortisone, and thyroxine replacement dose. At three-month follow-up, he is asymptomatic, the hyperpigmentation has diminished, and he has gained 8 kg of weight (Figure 4).

**Figure 5:**
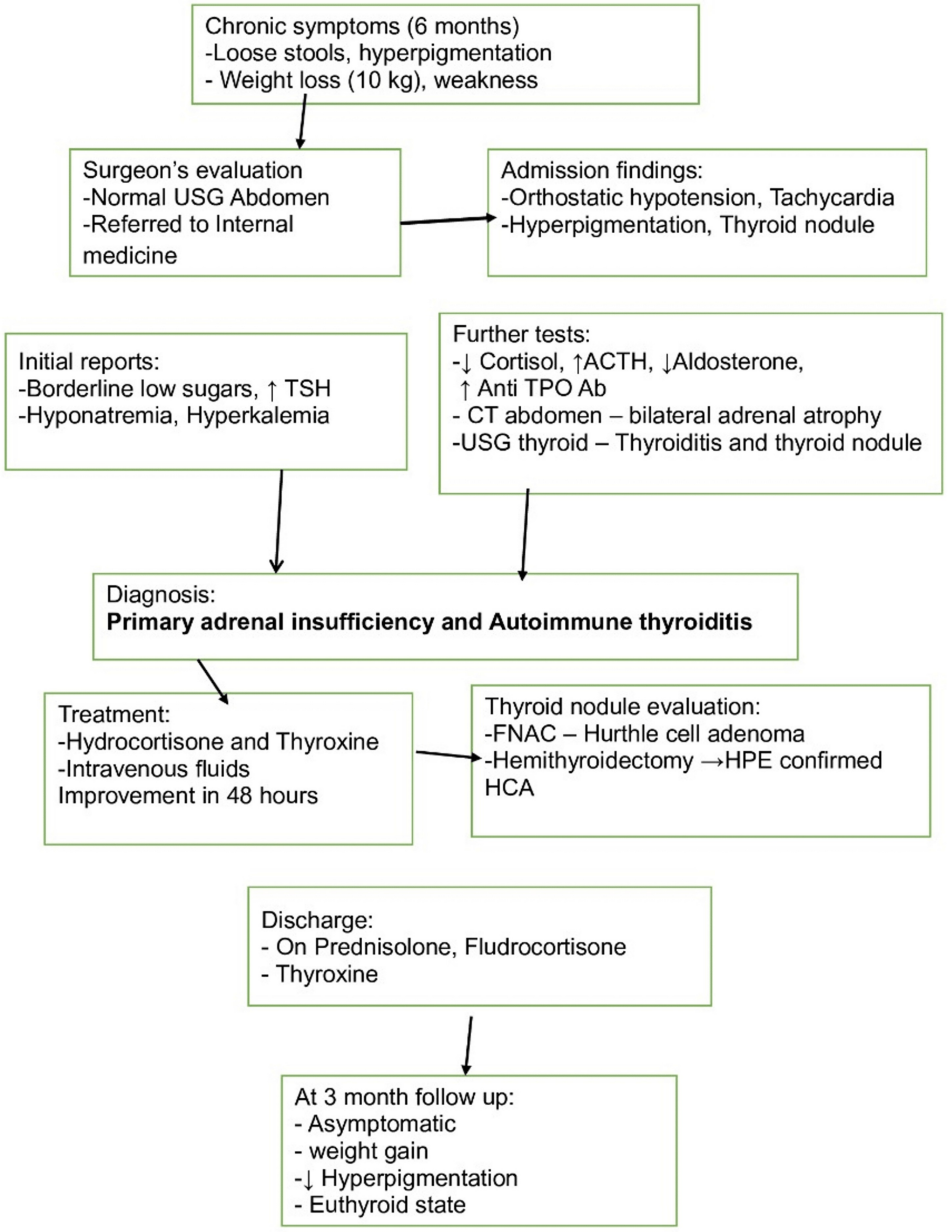
Schematic representation of clinical course, diagnosis, and management USG: ultrasonography, TSH: thyroid-stimulating hormone, ACTH: adrenocorticotropic hormone, anti-TPO Ab: anti-thyroid peroxidase antibodies, CT: computed tomography, FNAC: fine needle aspiration cytology, HPE: histopathological examination, HCA: Hurthle cell adenoma

The schematic representation of the clinical course, diagnosis, and management is given in Figure 5.

## Discussion

There are two major learning points from this case. The first is that a high index of suspicion is needed to diagnose not only primary adrenal insufficiency but also its being a part of APS 2 syndrome, and the second is the rare co-occurrence of HCA with APS 2.

Our patient presented with clinical and biochemical features of primary adrenal insufficiency, including hyperpigmentation, hyponatremia, hyperkalemia, orthostatic hypotension, borderline low blood sugars, and low cortisol with elevated adrenocorticotropic hormone. The computed tomography of the abdomen revealed bilateral adrenal atrophy. A test for 21-hydroxylase antibodies was not done in our patient in view of financial constraints. The thyroid function test was suggestive of subclinical hypothyroidism with high titers of anti-thyroid peroxidase antibodies, consistent with autoimmune thyroiditis, a major component of APS 2 [1-3].

The diagnosis of APS 2 is often missed or delayed in view of vague constitutional symptoms and asynchronous development of associated diseases. Primary adrenal insufficiency is a presenting disease in almost 50% of patients with APS 2 [2]. In APS 2, simultaneous presentation of primary adrenal insufficiency with autoimmune thyroiditis is a rarity, as in our case. The case reports by Upala et al. [5] and Lakhani et al. [6] have also shown simultaneous presentation of primary adrenal insufficiency and hypothyroidism. Unlike our case, in each one of them, hypothyroidism was diagnosed first, and when the patient did not respond to thyroxine treatment, primary adrenal insufficiency was considered.

The management of APS 2 involves the replacement of deficient hormones and careful sequencing of therapy. In our case, corticosteroid replacement was done first to avoid precipitating an adrenal crisis, followed by thyroid hormone therapy [5-7]. The thyroid nodule was surgically resected prophylactically as per the American Thyroid Association guidelines [8]. Postoperative follow-up showed significant clinical improvement with normalization of electrolyte balance, weight gain, and resolution of hyperpigmentation. Early diagnosis and prompt treatment are usually rewarding in patients with APS 2. A regular surveillance for the development of other associated diseases is recommended lifelong in these patients, and surveillance for autoimmune diseases in their siblings is mandatory [1]. Our patient’s siblings do not have any autoimmune diseases at present. It has been only a year since the diagnosis; relapse and development of any other associated diseases will require long-term follow-up.

Interestingly, in our patient, the thyroid examination revealed a solitary right thyroid nodule, confirmed on ultrasound and fine needle aspiration biopsy to be an HCA. As 30% of these tumors might progress to aggressive Hurthle cell carcinoma, hemithyroidectomy was done as per the recommendations by the American Thyroid Association [4,8,9], and subsequent histopathology confirmed HCA. It is difficult to differentiate between HCA and Hurthle cell carcinoma on fine needle biopsy; definitive diagnosis requires histopathological examination [4]. A small percentage of Hashimoto’s thyroiditis cases are known to develop into HCA over decades. The pathogenesis of Hurthle cell tumors in autoimmune thyroiditis is poorly understood. It is postulated that chronic autoimmune stimulation and reactive oxygen species generation may drive mitochondrial hyperplasia and oncocytic metaplasia [4,9]. These tumors are usually less than 4 cm in size, as was seen in our patient. The clinical course of HCA is indolent, with most remaining asymptomatic and growing slowly over many years. The overall prognosis is excellent, and there is no recurrence post-surgery [4,9]. Regular follow-up with thyroid function test and neck ultrasound is advocated to detect any late changes.

Molecularly, Hurthle cell tumors show alterations in mitochondrial DNA, especially common deletions and ATPase 6 gene polymorphisms. Chromosomal imbalances are more common in carcinoma than adenoma, especially losses on chromosome 22 [9]. Since histopathology confirmed HCA in our patient, genetic testing was deferred.

This case reinforces the importance of considering APS 2 in patients presenting with primary adrenal insufficiency and other subtle autoimmune features. Furthermore, the occurrence of HCA, although incidental, suggests the need for vigilance for neoplastic transformation in chronic autoimmune thyroid disease.

## Conclusions

This report highlights a rare presentation of APS 2 with Hurthle cell adenoma. In patients with primary adrenal insufficiency, it is important to evaluate for the presence of other coexisting autoimmune disorders. While autoimmune thyroiditis is a well-known component of APS 2, the association with a thyroid neoplasm is rare. Clinicians should maintain vigilance for structural thyroid abnormalities in such patients and consider surgical intervention when appropriate.
